# Primary thyroid angiosarcoma: an unusual localization

**DOI:** 10.1186/1477-7819-10-73

**Published:** 2012-05-03

**Authors:** Pasquale Petronella, Marco Scorzelli, Rossella Luise, Giuseppe Iannaci, Patrizia Sapere, Marco Ferretti, Rosaria Maria Anna Costanzo, Fulvio Freda, Silvestro Canonico, Raffaele Rossiello

**Affiliations:** 1 Department of Gerontology, Geriatry and Metabolic Diseases, UOC of General and Geriatric Surgery, School of Medicine, Second University of the Study of Naples, Piazza Miraglia, 80138, Naples, Italy; 2 Department of Public, Clinic and Preventive Medicine, Division of Pathology, School of Medicine, Second University of the Study of Naples, L. Armanni, 80138, Naples, Italy

**Keywords:** Angiosarcoma, Non-alpine region, Old man

## Abstract

The finding of thyroid nodules is a very common occurrence in routine clinical practice. Approximately 5% to 7% of the entire population have thyroid nodules. Vascular lesions are one of the most controversial issues in thyroid pathology. These include benign lesions such as hemangiomas and, rarely, malignant tumors such as angiosarcomas or undifferentiated angiosarcomatoid carcinomas. In particular, angiosarcoma of the thyroid gland is a rare, highly aggressive malignant vascular tumor and in Italy the greatest geographical incidence of this lesion is witnessed near the Alps. Here, a case of thyroid angiosarcoma in a 71-year-old man with a history of goiter for about 20 years is described. The unusual localization of this lesion, the difficulties in reaching a definitive diagnosis for this particular histological type of primary tumor and a history of long-standing multinodular goiter in thyroid of an older man from outside the Alpine region prompted us to report this case of thyroid angiosarcoma mainly to discuss surgical, histopathological and immunohistochemical features.

## Background

The finding of thyroid nodules is a very common occurrence in routine clinical practice. Approximately 5% to 7% of the entire population have thyroid nodules. The most common causes are benign conditions (colloid cysts and thyroiditis 80% of cases), followed by benign follicular neoplasias (10% to 15%) and carcinomas (5%) [1,2]. The majority of clinically apparent thyroid neoplasms are primary and epithelial, while mesenchymal tumors, which are commonly seen in other parts of the body, are rare in the thyroid [3,4].

Vascular lesions are one of the most controversial issues in thyroid pathology. These include benign lesions such as hemangiomas and, rarely, malignant tumors such as angiosarcomas or undifferentiated angiosarcomatoid carcinomas [5].

Angiosarcoma is, in general, an uncommon soft tissue sarcoma. This lesion mainly affects the skin and deep soft tissue but the involvement of the head and neck region is therefore usually reported in the literature, mainly in patients with a history of long-standing multinodular goiter. Location in the thyroid gland is nevertheless quite exceptional.

In particular, angiosarcoma of the thyroid gland is a rare and highly aggressive malignant vascular tumor [3,6-11]. The greatest incidence of this lesion is witnessed near the Alps. It constitutes only 2% to 10% of malignant thyroid tumors in Switzerland, Austria and Northern Italy [3,7,9].

The prevalence of this lesion in Alpine regions may be due to iodine deficiency with a long history of endemic goiter [8]. It has a female predilection (female to male ratio of 9:3) and arises in old age, most commonly during the fifth to eighth decades of life (median age 65 years) [7-9]. There are cases in which the tumor occurs without a history of goiter, and it is very unusual in non-Alpine areas, therefore it seemed prudent to present a case involving this type.

Most of these tumors appear as poorly encapsulated and infiltrating masses, which tend to grow in the absence of pain. Local recurrences and metastases are common, even after complete excision [6,12-14].

The prognosis of this tumor is not favorable because it tends to spread rapidly and most patients develop postoperative early systemic metastasis. In fact, this type of cancer typically metastasizes in the first instance at the level of regional lymph nodes and the lungs, and in the late stages in the bone marrow [12]. A very wide surgical excision is the management of choice in cases of radically removable tumor. Radiation therapy may be effective in some patients and can be completed using chemotherapy with adriamicina [6].

We describe a case of thyroid angiosarcoma in a 71-year-old man with a history of goiter for 20 years, who decided to undergo operation for worsening dyspnea. The unusual localization of this lesion, the difficulties in reaching a definitive diagnosis for this particular histological type of primary tumor and a history of long-standing multinodular goiter in the thyroid of an older man from outside the Alpine region prompted us to report this case of thyroid angiosarcoma mainly to discuss surgical, histopathological and immunohistochemical features.

## Case presentation

A 71-year-old man was referred to our department complaining of dyspnea, hypoventilation and dysphonia, determined by swelling in the neck region and related to a considerable increase in size of the thyroid gland.

On clinical examination, the thyroid gland appeared firm during the acts of deglutition. The patient reported that he had a goiter for more than 20 years and he had never undergone any drug therapy.

An ultrasound examination dating back to 1997 documented a complete subversion of the echotexture of the whole gland and the presence of a large nodule in the right lobe displaying a complex echotexture. A further ecography in 2000 documented an increase in volume of the thyroid, which was also the cause of the right carotid bulb dislocation.

Laboratory investigations revealed significantly elevated thyroglobulin values. A preoperative fine needle aspiration was not significant; it consisted of an acellular smear within a background of inflammatory and necrotic cells without any cytologic specification.

Consequently, we decided not to repeat this procedure because of the worsening of the patient’s dyspnea, for which a surgical management for liberation of the airways was planned.

**Figure 1:**
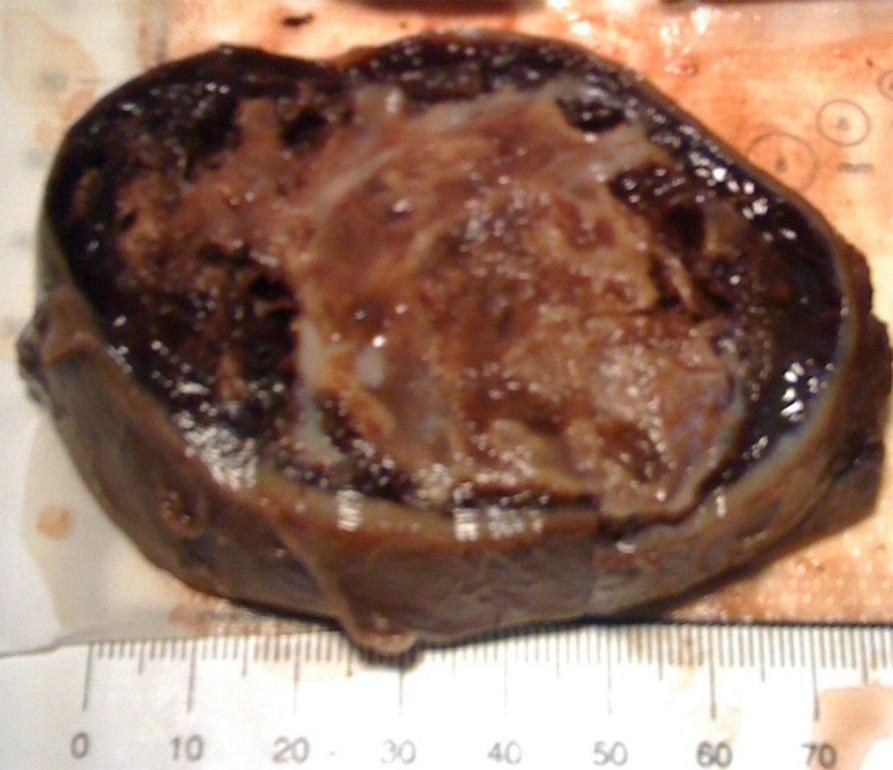
A macroscopic photograph of thyroid angiosarcoma showing a well capsulated nodule that appeared cystic and hemorrhagic on the cut surface.

A total thyroidectomy was performed. Although the surgical operation was expected to be very complex, it was performed in a completely linear way. The left lobe was easily separable from the surrounding tissue; it appeared to be in the throes of a nodular transformation. The right lobe appeared uniformly in a nodular transformation and it penetrated the upper part of the neck, adhering to the vessels, from which, however, it was easily dissociated. Lymph nodes were not visible. However, surgical times were also lower than expected, particularly for the removal of the right half, which was relatively easy, and the mass appeared well encapsulated and demarcated (Figure 1).

**Figure 2:**
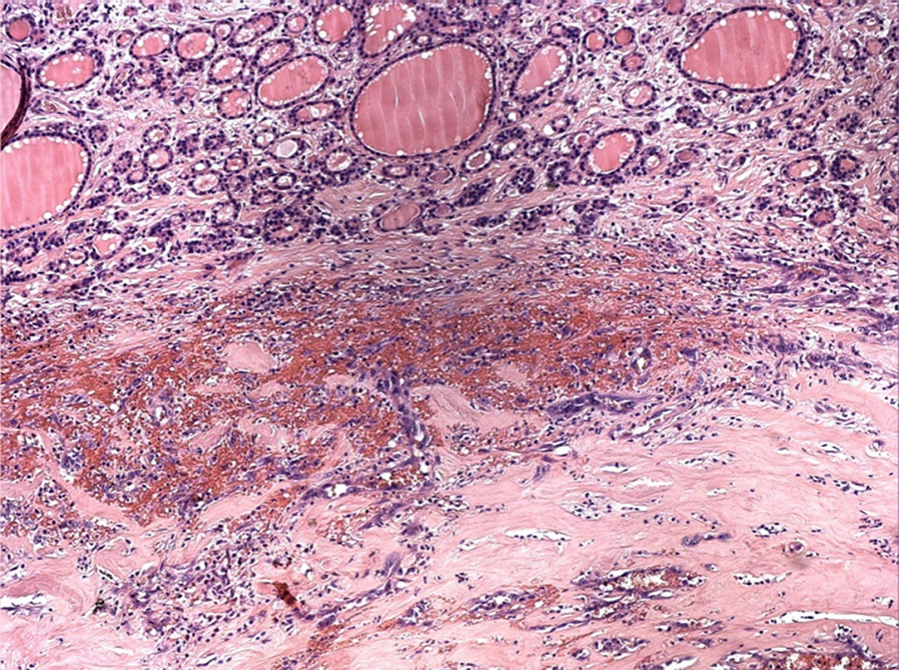
Hematoxylin and eosin staining (10 ×) shows a peripheral rim of normal thyroid tissue and a central area of necrosis and hemorrhages.

**Figure 3:**
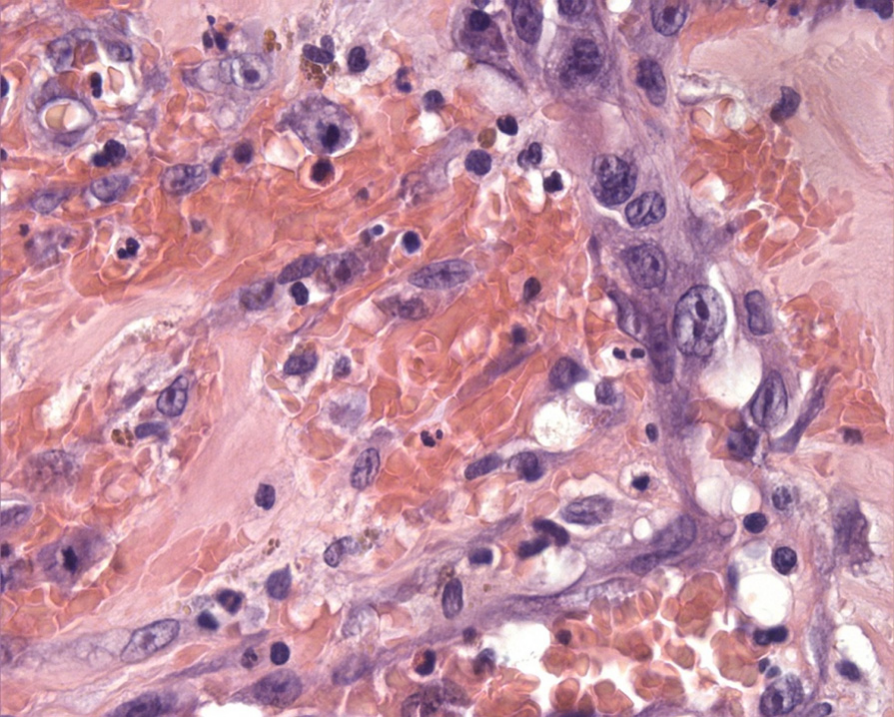
Hematoxylin and eosin staining (40 ×) showing numerous vascular channels lined by neoplastic endothelium.

**Figure 4:**
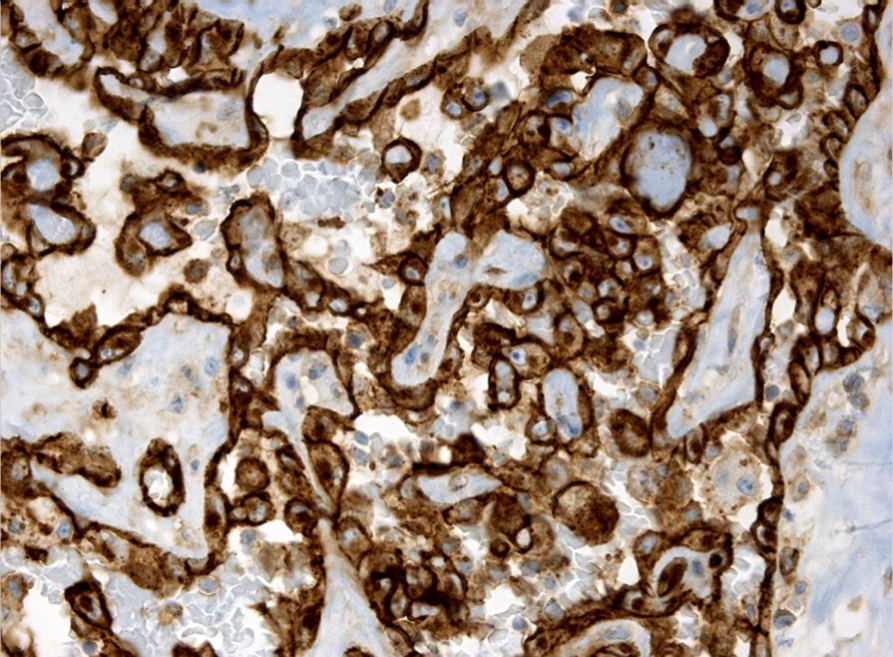
Vascular neoplastic channels with strong, diffuse CD 34 immunoreactivity.

The specimen was sent to surgical pathology for definitive diagnosis. The tissue samples were fixed in formalin, then routinely processed and embedded in paraffin. The sections were stained with hematoxylin and eosin. Additional 5-μm sections were cut and subjected to immunohistochemical studies using antibodies to the following antigens: CD34, CD31, factor VIII-related antigen, pan-cytokeratin (Pan-CK) and thyroglobulin. Grossly, the right thyroid lobe measured 10 × 8.5 × 6 cm, the left lobe was 5 × 4 × 1.5 cm and the pyramidal lobe was 4 × 2 × 1 cm in size. The right thyroid lobe was totally occupied by a well circumscribed nodule macroscopically confined within the capsule. The nodule measured 9 × 5 cm and, on the cut sections, appeared cystic and hemorrhagic, with large necrotic tissue areas (Figure 2). The tumor was extensively sampled. On histological examination, the periphery of the lesion showed epithelioid areas that were made up of large rounded cells of relatively high nuclear grade, with eosinophilic cytoplasm and prominent nucleoli arranged in rudimentary vascular channels. These neoplastic channels were irregular in shape, and they were lined by a single layer of malignant endothelium forming intraluminal papillary projections (Figure 3). An extensive central area of necrosis and hemorrhages was a characteristic feature of the lesion. The capsular surface was not involved with the tumor and a rim of residual thyroid tissue was observed in some peripheral areas. Immunohistochemically, neoplastic cells were strongly positive for CD31, CD34, and factor VIII-related antigen, showing evidence of their endothelial differentiation (Figure 4). Diagnosis of this condition can be difficult as the histological features may mimic other malignant vascular lesions. In view of the clinical history and morphological and immunohistochemical findings, a diagnosis of primary angiosarcoma of the thyroid gland was made.

As regards adjuvant therapy, our patient has not been subjected to any kind of special treatment because, even today, there are no guidelines and there are various schools of thought as to the best treatment options.

The prognosis was not favorable. The postoperative course was not simple: it was decided to admit the patient to our intensive coronary unit for precautionary reasons. After 3 days, due to an atelectasis of the left lung, intubation and subsequently tracheostomy were needed. Breathing difficulties continued for a long time (about 60 days after surgery). Our patient was monitored and follow-up was started but it was too short to be meaningful because unfortunately, our patient died about 3 months later as a result of of cardiac and respiratory complications related to his oncologic condition.

## Conclusions

Due to the cytologic and histologic presentation diagnosis of angiosarcoma of the thyroid gland is often difficult and requires an expert pathologist. Sometimes, on fine needle aspiration cytology (FNAC) samples, these vascular neoplasms yield a final report of ‘inadequate’ material or may closely mimic a number of different lesions, potentially causing an erroneous cytopathologic diagnosis [15,16]. For this reason, a definitive diagnosis of the angiosarcoma is based mainly on characteristic histopathological features of a malignant vascular tumor and must be supported by detailed investigations (immunopositivity for vascular markers for example, CD31, CD34, factor VIII related antigen, and absence of epithelial markers). However, care should be taken to distinguish a reactive process from vascular neoplasms such as hemangioma or angiosarcoma, because an exuberant endothelial cell proliferation may be a potential manifestation of a thyroid hematoma [17]. Cases, as in our patient, of long-standing nodular goiter with complete infarction are a real challenge for both the pathologist and the clinician/radiologist [5].

Moreover, the distinction between angiosarcoma and anaplastic sarcomatoid carcinoma is difficult and the same expression of the angiosarcoma has been subject to dispute [3,5,10,18].

Some authors controversially considered whether the thyroid angiosarcoma truly exists, because they are of the opinion that the reported cases were being classified as anaplastic carcinomas with angiomatoid features [5,18]. It may be that the angiosarcomas are ‘transitional’ tumors, that can show a variable appearance of mesenchymal metaplasia with both epithelial and endothelial differentiation. ‘Angiosarcoma’ may represent the extreme in the spectrum of endothelial differentiation. In fact, the WHO classification of thyroid tumors, published in 2004, has added to the four traditional major tumor groups (papillary, follicular, medullary, and anaplastic carcinoma) the entity of poorly differentiated carcinoma and a variety of rare thyroid malignancies, such as angiosarcoma [3]. At present, it must be acknowledged that the distinction between undifferentiated angiomatoid thyroid carcinomas and ‘true’ thyroid angiosarcomas is an academic one, because the prognoses and treatments for these lesions are essentially identical [18].

Their etiology remains unknown even though endothelial proliferations after recurrent intranodular hemorrhages occurring in a long-standing nodular goiter have been considered to initiate neoplastic transformation [3,19]. Immunohistochemical confirmation of the diagnosis of angiosarcoma, even those that are poorly differentiated, can usually be obtained using a panel of vascular markers. Antibody directed against CD31 is still considered the most sensitive and specific marker for endothelial differentiation, being expressed in 90% of angiosarcomas and in slightly more than 1% of carcinomas; the endothelial marker CD34 and the antigen related to factor VIII should also be added to the panel [10,20].

Immunonegativity for thyroglobulin supports a diagnosis of angiosarcoma. In fact, a weak signal for this antibody is seen in all anaplastic carcinomas, confirming that these lesions are unrelated malignant tumors [21]. The lack of staining with Pan-CK argues against an endothelial differentiation but there are cases reported in the literature that are interpreted as epithelioid angiosarcomas even in the presence of keratin positivity, a marker traditionally regarded as indicative of epithelial differentiation [19]. Finally, when faced with these lesions it is important to exclude metastasis from a more common primary site. Treatment is difficult because of its locally aggressive and destructive behavior, with a high recurrence rate. As regards adjuvant therapy, nowadays there exist various schools of thought. There are those who say that, after the radical excision of the tumor, radiotherapy and then chemotherapy are indicated [6,22]; others claim that, if the tumor is not corroded surgically, if the patient is subjected to radiotherapy and chemotherapy it represents only a palliative treatment because the first step of choice is radical surgical excision of the neoplasm, as seen in our patient [6,8].

The features that have been correlated with poor outcome include extracapsular tumor spread and distant metastasis [5-10]. Data on survival are sparse. With regard to prognosis, Goh *et al*. showed a 5-year survival rate of 33.3%. Most patients die in less than 6 months regardless of the treatment with a few surviving up to 5 years [5,9,13,14]. Entirely intrathyroid tumors generally have a longer survival than those with extrathyroidal extension [3,6-9]. However, cases of long survival have been reported, especially from non-mountainous, non-endemic goiter areas [3,9,23].

In conclusion, we present a rare case of primary thyroid angiosarcoma in a patient from a non-Alpine area with a history of long-standing goiter in the absence of pain and without evident signs of rapid growth, but with worsening dyspnea.

Despite a wide surgical excision and the lesion being limited to the thyroid gland, our patient died a few months after surgery; therefore the follow-up was not contributory to establishing the rate of disease-free survival.

We report and confirm the diagnosis even though the cytological and histopathological examination presented some difficulties in distinguishing it from other malignant tumor such as undifferentiated angiosarcomatoid carcinoma. Initial evaluation of hematoxylin and eosin stained sections and appropriate immunohistochemical panel for endothelial markers (CD31, CD34 and factor VIII) can usually provide a definitive diagnosis.

## Consent

Written informed consent was obtained from the patient for publication of this report and any accompanying images.

## Competing interests

The authors declare that they have no competing interests.

## Authors’ contributions

PP oversaw the entire clinical and surgical, contributed to the content of the manuscript and critically reviewed the manuscript. MS oversaw the entire clinical and surgical, contributed to the content of the manuscript and critically reviewed the manuscript. RL oversaw the histology, contributed to the content of the manuscript and critically reviewed the manuscript. GI oversaw the histology, contributed to the content of the manuscript and critically reviewed the manuscript. PS edited the histology, contributed to the content of the manuscript and critically reviewed the manuscript. MF oversaw the entire clinical and surgical, contributed to the content of the manuscript and critically reviewed the manuscript. RC oversaw the histology, contributed to the content of the manuscript and critically reviewed the manuscript. FF oversaw the entire clinical and surgical, contributed to the content of the manuscript and critically reviewed the manuscript. SC oversaw the entire clinical and surgical, contributed to the content of the manuscript and critically reviewed the manuscript. RR oversaw the histology, contributed to the content of the manuscript and critically reviewed the manuscript. All authors read and approved the final manuscript.
